# Text Messaging and Web-Based Survey System to Recruit Patients With Low Back Pain and Collect Outcomes in the Emergency Department: Observational Study

**DOI:** 10.2196/22732

**Published:** 2021-03-04

**Authors:** Anita Barros Amorim, Danielle Coombs, Bethan Richards, Chris G Maher, Gustavo C Machado

**Affiliations:** 1 Discipline of Physiotherapy, Sydney School of Health Sciences Faculty of Medicine and Health The University of Sydney Sydney Australia; 2 Institute for Musculoskeletal Health The University of Sydney and Sydney Local Health District Sydney Australia; 3 Physiotherapy Department Royal Prince Alfred Hospital Sydney Australia; 4 Rheumatology Department Royal Prince Alfred Hospital Sydney Australia

**Keywords:** emergency department, clinical trial, low back pain, acute pain, data collection, patient recruitment, short message service, patient reported outcome measures, mobile phone

## Abstract

**Background:**

Low back pain (LBP) is a frequent reason for emergency department (ED) presentations, with a global prevalence of 4.4%. Despite being common, the number of clinical trials investigating LBP in the ED is low. Recruitment of patients in EDs can be challenging because of the fast-paced and demanding ED environment.

**Objective:**

The aim of this study is to describe the recruitment and response rates using an SMS text messaging and web-based survey system supplemented by telephone calls to recruit patients with LBP and collect health outcomes in the ED.

**Methods:**

An automated SMS text messaging system was integrated into Research Electronic Data Capture and used to collect patient-reported outcomes for an implementation trial in Sydney, Australia. We invited patients with nonserious LBP who presented to participating EDs at 1, 2, and 4 weeks after ED discharge. Patients who did not respond to the initial SMS text message invitation were sent a reminder SMS text message or contacted via telephone. The recruitment rate was measured as the proportion of patients who agreed to participate, and the response rate was measured as the proportion of participants completing the follow-up surveys at weeks 2 and 4. Regression analyses were used to explore factors associated with response rates.

**Results:**

In total, 807 patients with nonserious LBP were invited to participate and 425 (53.0%) agreed to participate. The week 1 survey was completed by 51.5% (416/807) of participants. At week 2, the response rate was 86.5% (360/416), and at week 4, it was 84.4% (351/416). Overall, 60% of the surveys were completed via SMS text messaging and on the web and 40% were completed via telephone. Younger participants and those from less socioeconomically disadvantaged areas were more likely to respond to the survey via the SMS text messaging and web-based system.

**Conclusions:**

Using an SMS text messaging and web-based survey system supplemented by telephone calls is a viable method for recruiting patients with LBP and collecting health outcomes in the ED. This hybrid system could potentially reduce the costs of using traditional recruitment and data collection methods (eg, face-to-face, telephone calls only).

**International Registered Report Identifier (IRRID):**

RR2-10.1136/bmjopen-2017-019052

## Introduction

### Background

Low back pain (LBP) is a frequent reason for emergency department (ED) presentations, with a global prevalence of 4.4% [1]. This places LBP in the top 10 presenting complaints in the ED [2], as a broad spectrum of illnesses and injuries are seen in this setting. For comparison, the most common reason for visits to EDs (*injuries and adverse effects*) has a prevalence of 18% and the second most common reason (*cough, upper respiratory symptoms, or ears/nose/throat symptoms*) has a prevalence of 9% [3].

Despite being common, the number of clinical trials investigating LBP in emergency settings is surprisingly low [4]. One possible reason is the challenge in recruiting and collecting patient-reported outcomes in such a busy environment—EDs are often overcrowded, patients might present after hours, emergency clinicians do not have sufficient time, and there is a lack of administrative and structural support for research [5]. Employing effective and efficient methods for recruiting study participants and collecting data is crucial for optimizing research in this setting.

Mobile phones and internet-based technologies have been widely used to recruit and collect data for clinical trials in recent times [6,7]. Advantages regarding accessibility such as being low cost and time efficient and providing access to hard-to-reach populations make this method appealing for research [8]. There is also evidence showing that data collected via mobile phones and internet-based technologies are reliable, valid, and feasible [9,10]. For example, the use of mobile phone services, such as SMS text messaging, has been tested for data collection in LBP research in primary care, showing high response rates [11]. However, little is known about the use of SMS text messaging in combination with a web-based survey system to recruit participants and collect health outcomes in ED settings.

### Objectives

To explore the use of contemporary, low-cost, and more efficient options for conducting clinical research in EDs, we investigated the recruitment and response rates when using an SMS text messaging and web-based survey system, supplemented by telephone interviews, within a stepped-wedge cluster randomized controlled trial [12]. The primary aim of this study is to investigate the recruitment and response rates after an ED presentation for LBP. As previous research has suggested that participants’ characteristics, such as age, sex, and socioeconomic status, can significantly influence response rates [13], we also aim to investigate whether these factors influenced response rates in the ED setting.

## Methods

### Design

This is an observational study nested within a stepped-wedge cluster randomized controlled trial that evaluated the implementation of an evidence-based model of care for LBP in four public hospital EDs. The protocol for the Sydney Health Partners Emergency Department (SHaPED) trial has been published elsewhere [12]. The study design, procedures, and informed consent were approved by the Human Research Committees of the Sydney Local Health District (Royal Prince Alfred Hospital zone, protocol number X17-0043).

### Participants

Participants were recruited between July and December 2018 from the EDs of four public hospitals in New South Wales (NSW), Australia: Concord Repatriation General Hospital, Royal Prince Alfred (RPA) Hospital, Canterbury Hospital, and Dubbo Base Hospital. Eligible patients were identified using discharge diagnosis codes from the Systematized Nomenclature of Medicine—Clinical Terms—Australian version, Emergency Department Reference Set [14] (Multimedia Appendix 1). Only patients presenting to the EDs with nonserious forms of LBP (nonspecific LBP, sciatica, and lumbar spinal stenosis) and with a mobile phone number recorded in the medical records were invited to participate. Representations to the ED within 48 hours or LBP related to serious spinal pathologies, such as lumbar fracture, infection, malignancy, or cauda equina syndrome, were excluded.

### Recruitment

Upon discharge from the ED, eligible patients were informed about the study by clinical staff and/or received a flyer with information about a text message invitation and web-based survey. This method was used to ensure that the patients were aware of the survey before receiving the SMS text message invitation. Then, local clinical staff obtained patients’ information (ie, name, mobile number, and postcode) from the hospital’s electronic medical records. Patients’ information was inserted into a secure web app (Research Electronic Data Capture [REDCap]) by research staff.

An automated SMS text messaging system (Twilio Inc) was integrated into REDCap and used to schedule SMS text message invitations. We used an opt-out approach to ensure that there was no pressure or coercion on patients to consent to participate in the survey. Seven days after the ED visit at 12:30 PM, an SMS text message was sent to eligible patients with an invitation and link to answer the web-based survey. Patients who did not want to be invited to participate in the study could inform the local clinical staff at the time of discharge, who would then notify researchers to remove them from the invitation list. Potential participants could also ignore the SMS text message invitation or opt out by replying *NO* and would no longer be contacted. REDCap was scheduled to send an SMS text message (via Twilio) containing the following text approved by the Human Research Ethics Committee: “Dear [name], hope you are going well after your recent visit for back pain to our ED. We are interested in how your back is going and what you thought of our care. Our survey will take only 5 minutes to complete. This survey is being conducted by Dr [name], ED Director of [hospital]. To opt out reply NO -- to begin the survey, visit [link]”

The link in the SMS text message invitation referred eligible patients to the web-based participant information statement and consent information. At this point, potential participants could decline to participate and would no longer be contacted. Those who agreed to participate were referred to a brief self-reported web-based survey aimed at collecting patient-reported outcomes (Multimedia Appendix 2). Completion of the web-based survey indicated consent to participate in the survey. The survey was not anonymous, and patients did not receive any financial remuneration for responding to it.

For those who agreed to participate and completed the week 1 survey, 2 follow-up surveys were sent at 2 and 4 weeks after ED presentation. Initially, we scheduled reminder messages to be sent 3 times (on consecutive days) for each data collection wave if the survey had not been completed or the patient had not opted out of the study. Therefore, each participant had up to four opportunities to respond to the web-based survey directly on their smartphone. Halfway through the study, we changed the scheduling system to one reminder only, as suggested by our Human Research Ethics Committee, to avoid patients becoming overwhelmed by the number of text message invitations. To maximize the response rate, participants who did not respond to the final reminder message were contacted verbally via telephone. Not completing the week 2 survey, either on the web or via telephone, did not preclude participants from completing the week 4 survey. The research staff followed a script approved by the Human Research Ethics Committee to conduct the telephone survey.

### Predictors and Outcomes

In this study, we investigated recruitment and response rates as outcomes. Patients’ responses to 3 surveys (ie, at 1, 2, and 4 weeks after discharge) were classified as *yes* (survey completed) or *no* (survey not completed or the patient opted out of the study). We also classified (yes/no) whether responses occurred at the initial SMS text message invitation, after reminder messages, or during telephone calls. Recruitment rates were measured as the proportion of eligible patients consenting to participate upon first SMS text message invitation, after a reminder SMS text message, or during telephone calls (yes/no). Response rates were measured as the proportion of included participants completing the follow-up surveys at weeks 2 and 4 via initial SMS text message invitation, reminder SMS text message, or telephone calls (yes/no).

We had limited access to patient’s information and extracted the following putative predictors from the hospital’s electronic medical records: age, sex, and postcode. The Australian Bureau of Statistics’ Socio-Economic Indexes for Areas (SEIFA) 2016 [15] was used to classify patients’ socioeconomic status based on their postcode of residence. SEIFA was reported as deciles, with the lowest decile designating areas with the greatest socioeconomic disadvantage.

### Analyses

Descriptive analyses were used to report recruitment and response rates (via initial SMS text message, reminder SMS text message, or telephone calls) for all participants and grouped by recruitment site, age, sex, and socioeconomic status. Recruitment and response rates were also calculated for each month during the 6-month trial period. A multiple logistic regression model was used to evaluate whether age, sex, and socioeconomic status (SEIFA deciles) were predictors of whether a participant responded to the survey on the web or via telephone calls. In contrast to the other 3 metropolitan Sydney sites included in the parent study, Dubbo Base Hospital is located in a rural area of NSW. Thus, we decided to analyze whether there were any differences in recruitment or response rates between the metropolitan and rural EDs. All data analyses were conducted using STATA (version 14.0, STATA Corporation).

## Results

### Recruitment Rate

**Figure 1:**
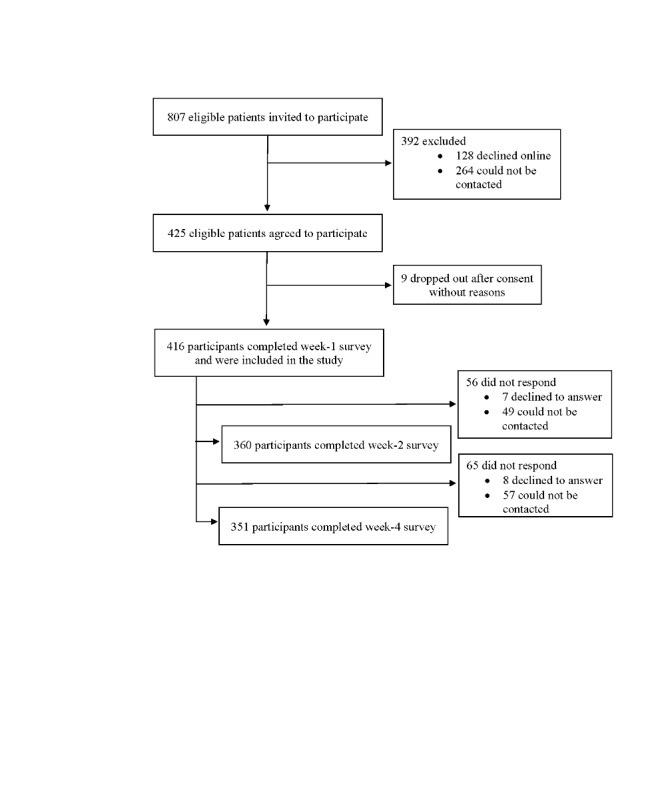
Study flowchart.

Data were collected between July and December 2018. In total, 807 eligible patients with nonserious LBP were invited to participate and 425 (53.0%) agreed to participate. After 9 participants dropped out without reasons, 51.5% (416/807) entered the study and completed the week 1 survey. At week 2, 86.5% (360/416) participants completed the follow-up survey, and at week 4, 84.4% (351/416) completed the survey (Figure 1). The highest recruitment rates were in females (212/392, 54.1%), at Concord Hospital (113/195, 57.9%), among people aged 40 to 69 years (83/141, 58.9% - 68/109, 62.4%), and from people living in the least socioeconomic disadvantaged areas (61/107, 57.0% - 35/58, 60.3%—SEIFA deciles 8, 9, and 10; Table 1). Overall, 59.6% (248/416) of participants were recruited via SMS text messaging alone (including the reminder SMS text message) and 40.4% (168/416) agreed to participate via telephone calls.

**Table 1:** Recruitment rates by response method, grouped by recruitment site, age, sex, and socioeconomic status (N=807).

Variable		Invited to participate	Recruited via initial SMS text message	Recruited via reminder SMS text message	Recruited via telephone calls	Total recruited participants
Participant, n (%)		807 (100)	138 (17.1)	110 (13.6)	168 (20.8)	416 (51.5)
**Recruitment site, n (%)**						
	RPA^a^	292 (36.1)	70 (23.9)	42 (14.4)	46 (15.8)	158 (54.1)
	Canterbury	196 (24.3)	24 (12.2)	24 (12.2)	47 (23.9)	95 (48.5)
	Concord	195 (24.2)	35 (17.9)	27 (13.8)	51 (26.2)	113 (57.9)
	Dubbo	124 (15.4)	9 (7.3)	17 (13.6)	24 (19.2)	50 (40.3)
**Age group (years), n (%)**						
	18-29	123 (15.2)	14 (11.4)	17 (13.8)	18 (14.6)	49 (39.8)
	30-39	158 (19.6)	29 (18.3)	19 (12.0)	28 (17.7)	76 (48.1)
	40-49	141 (17.5)	26 (18.4)	27 (19.1)	30 (21.3)	83 (58.9)
	50-59	134 (16.6)	27 (20.1)	22 (16.4)	30 (22.4)	79 (59.0)
	60-69	109 (13.5)	26 (23.8)	10 (9.2)	32 (29.4)	68 (62.4)
	70-79	84 (10.4)	11 (13.1)	6 (7.1)	21 (25.0)	38 (45.2)
	80+	57 (7.1)	5 (8.8)	9 (15.8)	9 (15.8)	23 (40.0)
**Sex, n (%)**						
	Female	392 (48.6)	72 (18.4)	62 (15.8)	78 (19.9)	212 (54.1)
	Male	415 (51.4)	66 (15.9)	48 (11.6)	90 (21.7)	204 (49.2)
**Socioeconomic status (SEIFA^b^ deciles), n (%)**						
	1^c^	41 (5.1)	9 (21.9)	3 (7.3)	7 (17.1)	19 (46.3)
	2	39 (4.8)	5 (12.8)	6 (15.4)	10 (25.6)	21 (53.8)
	3	79 (9.8)	8 (10.1)	11 (13.9)	22 (27.8)	41 (51.9)
	4	124 (15.4)	12 (9.7)	16 (12.9)	23 (18.5)	51 (41.1)
	5	4 (0.5)	0 (0.0)	2 (50.0)	0 (0.0)	2 (50.0)
	6	68 (8.4)	13 (19.1)	10 (14.7)	10 (14.7)	33 (48.5)
	7	81 (10.0)	11 (13.5)	10 (12.3)	18 (22.2)	39 (48.1)
	8	58 (7.2)	13 (22.4)	10 (17.2)	12 (20.7)	35 (60.3)
	9	107 (13.3)	24 (22.4)	13 (12.1)	24 (22.4)	61 (57.0)
	10	206 (25.5)	43 (20.9)	29 (14.1)	42 (20.4)	114 (55.3)

^a^RPA: Royal Prince Alfred.

^b^SEIFA: Socio-Economic Indexes for Areas.

^c^Decile 1 contains the most disadvantaged areas.

### Response Rate

Table 2 shows the characteristics of the study participants (n=416) and the response rates at weeks 2 and 4. Participants’ mean age was 50.0 years (SD 17.1), 51% were female, most participants (38%) presented at the RPA Hospital ED, and half (50%) were from the least socioeconomic disadvantaged areas (SEIFA deciles 8, 9, and 10). The highest response rates at week 2 were at RPA Hospital (90%), among people aged 50 to 59 years (96%), females (87%), and those from the least socioeconomic disadvantaged areas (SEIFA deciles 8 [100%] and 10 [93%]). In contrast, response rates at week 4 were higher among older people (aged ≥80 years, 96%) and males (88%); however, similar rates were found in the other categories.

**Table 2:** Participants’ characteristics and response rates at weeks 2 and 4, grouped by recruitment site, age, sex, and socioeconomic status (n=416).

Variables		Total^a^	Responded at week 2^b^	Responded at week 4^b^
Participant, n (%)		416 (100)	360 (86.5)	351 (84.4)
**Recruitment site, n (%)**				
	RPA^c^	158 (38.0)	142 (89.9)	142 (89.9)
	Canterbury	95 (22.8)	80 (84.2)	76 (80.0)
	Concord	113 (27.2)	96 (85.0)	88 (77.9)
	Dubbo	50 (12.0)	42 (84.0)	45 (90.0)
**Age group (years), n (%)**				
	18-29	49 (11.7)	35 (71.4)	33 (67.3)
	30-39	76 (18.3)	64 (84.2)	62 (81.6)
	40-49	83 (19.9)	69 (83.1)	68 (81.9)
	50-59	79 (19.0)	76 (96.2)	71 (89.9)
	60-69	68 (16.4)	61 (89.7)	63 (92.6)
	70-79	38 (9.1)	34 (89.5)	32 (84.2)
	80+	23 (5.6)	21 (91.3)	22 (95.6)
**Sex, n (%)**				
	Female	212 (51.0)	185 (87.3)	171 (80.7)
	Male	204 (49.0)	175 (85.8)	180 (88.2)
**Socioeconomic status (SEIFA^d^ deciles), n (%)**				
	1^e^	19 (4.6)	17 (89.5)	14 (73.7)
	2	21 (5.0)	17 (80.9)	18 (85.7)
	3	41 (9.9)	34 (82.9)	34 (82.9)
	4	51 (12.3)	42 (82.3)	44 (86.2)
	5	2 (0.5)	1 (50.0)	1 (50.0)
	6	33 (7.9)	30 (90.9)	28 (84.8)
	7	39 (9.4)	33 (84.6)	31 (79.5)
	8	35 (8.4)	35 (100.0)	34 (97.1)
	9	61 (14.5)	45 (73.8)	51 (83.6)
	10	114 (27.4)	106 (93.0)	96 (84.2)

^a^Study sample characteristics at baseline. Percentages correspond to the number of participants in each group divided by the total number of participants.

^b^Response rates at weeks 2 and 4 grouped by recruitment site, age, sex, and socioeconomic status.

^c^RPA: Royal Prince Alfred.

^d^SEIFA: Socio-Economic Indexes for Areas.

^e^Decile 1 contains the most disadvantaged areas.

### Recruitment and Response Rates by Response Method

Table 3 shows recruitment and response rates by each study period and method of response (SMS text messaging and web-based system or telephone calls). Overall, recruitment rates varied during the study from 43% (December 2018) to 61% (September 2018). Response rates via the SMS text messaging and web-based system decreased over time (from 73% to 51%), whereas those via telephone calls increased from 27% to 49% during the study period. The results of a multiple logistic regression model presented in Table 4 show that web-based responses were significantly higher in those who were younger (odds ratio [OR] 0.99, 95% CI 0.97-0.99; *P*=.02) and those in less socioeconomic disadvantaged areas (OR 1.08, 95% CI 1.00-1.16; *P*=.03).

**Table 3:** Recruitment and response rates by response method, grouped by study period.

Study period (month)	Invited to participate, n	Recruited participants^a^, n (%)	Responded via SMS text message (initial and reminder)^b^, n (%)	Responded via telephone^b^, n (%)
July 2018	147	70 (47.6)	51 (72.9)	19 (27.1)
August 2018	146	74 (50.7)	56 (75.7)	18 (24.3)
September 2018	116	71 (61.2)	35 (49.3)	36 (50.7)
October 2018	154	82 (53.2)	45 (54.9)	37 (45.1)
November 2018	157	82 (52.2)	42 (51.2)	40 (48.8)
December 2018	87	37 (42.5)	19 (51.4)	18 (48.6)
Total	807	416 (51.5)	248 (59.6)	168 (40.4)

^a^Percentages based on the number of people invited to participate in the study.

^b^Percentages based on the number of people included in the study.

**Table 4:** Associations of age, sex, and socioeconomic status with response method (SMS text messaging and web-based or telephone).

Variables	SMS text messaging and web-based system at week 1 (n=416)		SMS text messaging and web-based system at week 2 (n=360)		SMS text messaging and web-based system at week 4 (n=351)	
	OR^a^ (95% CI)	*P* value	OR (95% CI)	*P* value	OR (95% CI)	*P* value
Age	0.99 (0.97-0.99)	.02	0.99 (0.98-1.00)	.17	1.00 (0.99-1.01)	.92
Sex	0.73 (0.49-1.08)	.12	0.79 (0.52-1.22)	.29	0.74 (0.48-1.13)	.16
Socioeconomic status	1.06 (0.99-1.13)	.08	1.08 (1.00-1.16)	.03	1.03 (0.96-1.11)	.40

^a^OR: odds ratio.

### Recruitment and Response Rates by Hospital Site

The overall recruitment rate in rural hospitals (50/124, 40.3%) was 13% lower than that in the metropolitan hospitals (366/683, 53.6%). Recruitment rates via SMS text message invitations differed substantially between rural (26/124, 20.9%) and metropolitan (222/683, 32/5%) areas but were similar for telephone calls (24/124, 19.4% and 144/683, 21.1%, respectively). Overall, at week 1, 59.6% (248/416) participants completed the study invitation and survey via the SMS text messaging and web-based system—33.1% (138/416) after the initial SMS text message invitation and 26.4% (110/416) after the reminders—and 40.4% (168/416) completed the survey over the telephone. At week 2, 61.1% (220/360) participants completed the survey via the SMS and web-based system—36.9% (133/360) after initial SMS text message invitation and 24.2% (87/360) after reminders—and 38.8% (140/360) completed the survey over the telephone. At week 4, 55.8% (196/351) participants completed the survey via the SMS text messaging and web-based system—30.5% (107/351) after the initial SMS text message invitation and 25.4% (89/351) after reminders—and 44.2% (155/351) completed the survey over the telephone.

### Overall Study Response Rate

The scheduling system changed from 3 reminders to 1 reminder in the first week of October 2018. After the change, the recruitment rate in the study was only 2% smaller, from 53% in July to September 2018 to 51% in October to December 2018. Responses via SMS text messaging (initial or reminder) had an absolute reduction of 13% (from 66% to 53%) at week 1, 7% (from 65% to 58%) at week 2, and 11% (from 61% to 50%) at week 4. The overall response rate (SMS text messaging plus telephone) was approximately 5% greater (84% vs 89%) at week 2 but 3% lower (86% vs 83%) at week 4 after changing the scheduling system to 1 SMS text message reminder (Multimedia Appendix 3).

## Discussion

### Principal Findings

Recruitment of patients in research, especially in emergency settings, remains a challenge for many studies because of the fast-paced, demanding environment of EDs. This study explored the recruitment and response rates when using an automated SMS text message (Twilio) in combination with a web-based survey system (REDCap) and/or telephone calls to recruit participants and collect LBP outcomes in the ED. Approximately half of all eligible patients were successfully recruited to the trial, and follow-up response rates ranged from 84% to 86%. This suggests that using an SMS text messaging and web-based survey system supplemented by telephone calls is a viable method for recruitment and data collection in this setting. Younger participants and those living in the least socioeconomically disadvantaged areas were more likely to complete the survey on the web rather than via telephone. Recruitment rates, particularly those via SMS text message invitation, were higher in metropolitan hospitals than in rural sites. These findings are similar to other studies that have shown that older people and those from more socioeconomically disadvantaged areas are less comfortable using smartphones for research purposes [16-18]. The response rates to the SMS text messaging and web-based methods alone at weeks 2 (61%) and 4 (56%) were noticeably lower than the desirable 85% follow-up rate, which was considered adequate for a clinical trial [19]. Consequently, it appears that SMS text messaging and web-based methods alone may not be practical for use as a substitute for traditional methods (eg, face-to-face, telephone calls) of follow-up contact in ED trials. However, we found that adding telephone calls to an automated SMS text messaging and web-based system of data collection increased response rates by up to 86%. Therefore, the use of a hybrid system (SMS text messaging and web-based survey plus telephone follow-ups), in which traditional methods of data collection are only used for those who do not respond to the automated SMS text messaging and web-based system, is a practical option and could potentially result in significant savings in cost and time.

Poor patient recruitment and response are two well-known threats to feasibility in clinical trials [20], especially in EDs because of the demanding and pressurized workplace [21]. These threats can lead to a considerable waste of financial resources or underpowered studies that report on clinically relevant research questions with insufficient statistical power. Therefore, establishing optimal methods to enhance recruitment and response rates in this particular setting is crucial. Although several recent clinical trials have used mobile technology, such as SMS text messaging, to collect outcomes [22], this study is the first to report the recruitment and response rates when using this approach to recruit patients with LBP and collect outcomes in an ED setting.

Our data support the findings of a similar study conducted by Macedo et al [11], demonstrating that SMS text messaging supplemented with telephone follow-ups, but not SMS text messaging alone, provides excellent follow-up response rates for randomized controlled trials with people with LBP. In contrast to our study, Macedo et al [11] investigated response rates in a cohort of patients who had already been included in the study via traditional face-to-face methods. Thus, this study is the first in the LBP field to examine recruitment rates via SMS text messaging as the primary invitation method. We also demonstrated the recruitment and response rates of patients in EDs using this approach, which have not been previously reported. On the basis of our findings, the use of a hybrid system (SMS text messaging and web-based survey plus telephone follow-ups) is a practical option in this setting and could provide considerable reductions in the costs of recruitment and data collection, as the costly traditional methods would only need to be used for approximately 41% of web-based nonresponders.

Considering the barriers involved in recruitment and follow-up assessment in ED trials and the potential advantages of using an SMS text messaging and web-based system to facilitate this, future studies should focus on methods to enhance compliance with these novel technologies. This is particularly important among older people and those from socioeconomically disadvantaged areas, as our results revealed that these factors were associated with lower response rates via web-based systems. In addition, future studies could perform cost-effective analysis comparing automated web-based systems with traditional methods and the benefits in reducing costs and increasing response rates when using a hybrid method. Future research should also explore patients’ experiences using this method.

### Limitations

Australia is one of the leading users of smartphones, with 89% of the population owning one, and, surprisingly, market growth is being driven by older generations [23]. The same is true for other high-income countries, such as Norway, the Netherlands, and the United Kingdom [24]. However, in low-income and middle-income countries, the scenario is different, with only approximately 45% of the population owning a smartphone, and only a minor proportion of these are owned by older people [25]. Therefore, generalization of our findings may be limited in those countries. Generalization of our results may also be limited to other health jurisdictions, as participants were predominantly recruited from 2 local health districts in NSW, Australia. Although our participants comprised individuals from diverse age groups and socioeconomic areas, we had limited information on other demographic and clinical characteristics. Another limitation is that when participants did not respond to a round of SMS text messaging, they were still contacted via telephone. Although this may have influenced our overall response rate, we analyzed the response rates separately for each method. Furthermore, in the first half of the trial period, participants received up to 3 reminders to complete the survey, which may be the reason for the increased response rate via the SMS text messaging and web-based system compared with telephone calls during this period.

### Conclusions

This study demonstrates that an automated SMS text messaging and web-based system in addition to telephone calls, but not SMS text messaging and web-based systems alone, is a viable option for recruiting patients with LBP and collecting outcome data in ED settings in Australia. This hybrid method is likely to facilitate recruitment and data collection in clinical trials in EDs and potentially reduce the costs of using traditional recruitment and data collection methods. However, generalization of our findings may be limited in the countries with a high percentage of smartphone use, such as the United States, Spain, and Germany, where smartphone ownership rates range from 85% to 89%. Future cost-effective analysis should be conducted in similar studies to allow for a clear conclusion regarding the potential cost savings of this method.

## Appendix

Multimedia Appendix 1 Systematized Nomenclature of Medicine—Clinical Terms—Australian version, Emergency Department Reference Set codes related to nonserious low back pain presentations.

Multimedia Appendix 2 Patient survey.

Multimedia Appendix 3 Differences in the recruitment and response rates throughout the study when sending the SMS text message reminder 3 times versus 1 time.

## Abbreviations

ED: emergency department
LBP: low back pain
NHMRC: National Health and Medical Research Council
NSW: New South Wales
OR: odds ratio
REDCap: Research Electronic Data Capture
SEIFA: Socio-Economic Indexes for Areas
SHaPED: Sydney Health Partners Emergency Department
