# Supplementary material for: Text Messaging and Web-Based Survey System to Recruit Patients With Low Back Pain and Collect Outcomes in the Emergency Department: Observational Study
Source: JMIR Mhealth Uhealth. 2021 Mar 4;9(3):e22732. doi: 10.2196/22732 (PMC7974753; doi:10.2196/22732)
Supplement: Multimedia Appendix 1 [file mhealth_v9i3e22732_app1.pdf]

Supplementary Appendix 1. SNOMED CT-AU (EDRS) codes related to non-serious low back pain presentations.

DESCRIPTION	CODES
Low back pain with non-specific cause	
Acute low back pain (finding)	278862001
Back pain complicating pregnancy (disorder)	91957002
Backache (finding)	161891005
Blunt injury to back (disorder)	424270008
Chronic back pain (finding)	134407002
Chronic low back pain (finding)	278860009
Coccyx sprain (disorder)	209571002
Complaining of low back pain (finding)	161894002
Degeneration of lumbar intervertebral disc (disorder)	26538006
Displacement of lumbar intervertebral disc without myelopathy (disorder)	20021007
Exacerbation of backache (finding)	135860001
Low back pain (finding)	279039007
Low back strain (disorder)	300956001
Lower back injury (disorder)	282766005
Lumbar spondylosis (disorder)	239880009
Lumbar sprain (disorder)	209565008
Mechanical low back pain (finding)	279040009
Pain in the coccyx (finding)	34789001
Sacral back pain (finding)	61486003
Spasm of back muscles (finding)	203095000
Sprain of ligament of lumbosacral joint (disorder)	209548004
Stiff back (finding)	249921008
Strain of back muscle (disorder)	262965006
Strain of tendon of back (disorder)	262975009
Low back pain with neurological signs and symptoms	
Acute back pain with sciatica (finding)	247366003
Acute sciatica (disorder)	307176005
Chronic sciatica (disorder)	307177001
Injury of lumbar nerve roots (disorder)	24300005
Injury of sciatic nerve (disorder)	86269002
Lumbago with sciatica (finding)	202794004
Lumbago-sciatica due to displacement of lumbar intervertebral disc (disorder)	46960006
Lumbar disc prolapse with radiculopathy (disorder)	202735001
Lumbar radiculopathy (disorder)	128196005
Sciatica (disorder)	23056005
Spinal stenosis of lumbar region (disorder)	18347007

SNOMED CT-AU (EDRS), Systematized Nomenclature of Medicine – Clinical Terms – Australian Version (Emergency Department Reference Set).
