# Supplementary material for: Text Messaging and Web-Based Survey System to Recruit Patients With Low Back Pain and Collect Outcomes in the Emergency Department: Observational Study
Source: JMIR Mhealth Uhealth. 2021 Mar 4;9(3):e22732. doi: 10.2196/22732 (PMC7974753; doi:10.2196/22732)
Supplement: Multimedia Appendix 2 [file mhealth_v9i3e22732_app2.pdf]

Supplementary Appendix 2. Patient Survey

Questions sent on weeks 1, 2 and 4

1. How would you rate your average low back pain intensity over the last week?

☐	☐	☐	☐	☐	☐	☐	☐	☐	☐	☐
0	1	2	3	4	5	6	7	8	9	10
No Pain										Worst imaginable Pain

	Without any difficulty	With a little difficulty	With some difficulty	With much difficulty	Unable to do
**2.** Are you able to do chores such as vacuuming or yard work?	☐ 5	☐ 4	☐ 3	☐ 2	☐ 1
**3.** Are you able to go up and down stairs at a normal pace?	☐ 5	☐ 4	☐ 3	☐ 2	☐ 1
**4.** Are you able to go for a walk of at least 15 minutes?	☐ 5	☐ 4	☐ 3	☐ 2	☐ 1
**5.** Are you able to run errands and shop?	☐ 5	☐ 4	☐ 3	☐ 2	☐ 1

	Excellent	Very good	Good	Fair	Poor
**6.** In general, would you say your health is:	☐ 5	☐ 4	☐ 3	☐ 2	☐ 1

Additional question sent at week 1 only

7. Using any number from 0 to 10, where 0 is the worst care possible and 10 is the best care possible, what number would you use to rate your care during this emergency room visit?

☐	☐	☐	☐	☐	☐	☐	☐	☐	☐	☐
0	1	2	3	4	5	6	7	8	9	10
Worst care possible										Best care possible
