# Supplementary material for: Text Messaging and Web-Based Survey System to Recruit Patients With Low Back Pain and Collect Outcomes in the Emergency Department: Observational Study
Source: JMIR Mhealth Uhealth. 2021 Mar 4;9(3):e22732. doi: 10.2196/22732 (PMC7974753; doi:10.2196/22732)
Supplement: Multimedia Appendix 3 [file mhealth_v9i3e22732_app3.docx]

| **Week 1** | | | | | | | |
| --- | --- | --- | --- | --- | --- | --- | --- |
|  | **Invited** | **Recruited** | **Refused** | **SMS (first)** | **SMS (reminder)** | **SMS (all)** | **Telephone** |
| Jul-Sep | 409 | 215 (53%) | 194 (47%) | 76 (35%) | 66 (31%) | 142 (66%) | 73 (34%) |
| Oct-Dec | 398 | 201 (50%) | 197 (50%) | 62 (31%) | 44 (22%) | 106 (53%) | 95 (47%) |
| Total | 807 | 416 (52%) | 391 (49%) | 138 (33%) | 110 (26%) | 248 (60%) | 168 (40%) |
| **Week 2** | | | | | | | |
|  | **Invited** | **Responded** | **No Response** | **SMS (first)** | **SMS (reminder)** | **SMS (all)** | **Telephone** |
| Jul-Sep | 215 | 181 (84%) | 34 (16%) | 75 (41%) | 42 (23%) | 117 (65%) | 64 (35%) |
| Oct-Dec | 201 | 179 (89%) | 22 (11%) | 58 (32%) | 45 (25%) | 103 (58%) | 76 (43%) |
| Total | 416 | 360 (87%) | 56 (14%) | 133 (37%) | 87 (24%) | 220 (61%) | 140 (39%) |
| **Week 3** | | | | | | | |
|  | **Invited** | **Responded** | **No Response** | **SMS (first)** | **SMS (reminder)** | **SMS (all)** | **Telephone** |
| Jul-Sep | 215 | 185 (86%) | 30 (14%) | 61 (33%) | 52 (28%) | 113 (61%) | 72 (39%) |
| Oct-Dec | 201 | 166 (83%) | 35 (17%) | 46 (28%) | 37 (22%) | 83 (50%) | 83 (50%) |
| Total | 416 | 351 (84%) | 65 (16%) | 107 (30%) | 89 (25%) | 196 (56%) | 155 (44%) |

**Appendix 3.** Differences in the recruitment and response rate throughout the study when sending the SMS reminder three times versus to one time.
